# Prevalence of Somatic Symptoms and Quality of Life Among Married Women Having a Distant Relationship With Their Spouses in Mangalore, India

**DOI:** 10.7759/cureus.21192

**Published:** 2022-01-13

**Authors:** Sumitha Pereira, Thereza Mathias, Shivakumara J, Jaison Jacob

**Affiliations:** 1 Mental Health Nursing, Tejaswini College of Nursing, Mangalore, IND; 2 College of Nursing, Laxmi Memorial College of Nursing, Mangalore, IND; 3 Mental Health Nursing, Laxmi Memorial College of Nursing, Mangalore, IND; 4 College of Nursing, All India Institute of Medical Sciences, Bhubaneswar, Khordha, IND

**Keywords:** left behind women, distant relationship, married women, quality of life, somatic disorders

## Abstract

Background: Married women having a distant relationship with their husbands are prone to mental disorders like somatic symptoms, eventually reducing their quality of life (QoL).

Objective: To assess the somatic symptoms and quality of life among married women having a distant relationship with their spouses.

Methodology: A descriptive cross-sectional study was conducted in 2018. Women having a distant relationship were selected using snowball sampling from selected urban areas of Mangalore, Karnataka. The Scale for Assessment of Somatic Symptoms (SASS) and the World Health Organization Quality of Life Scale (WHOQOL)-BREF questionnaire were used to assess somatic symptoms and quality of life, respectively. Multiple linear regression was used to identify the predictors of somatic symptoms and QoL.

Results: Out of 100 married women with a distant relationship, 83% experienced at least one symptom at a moderate/severe level, whereas the prevalence of somatic symptoms was 30% (at least one symptom at a severe level). Among these 30 women, the majority were in the age groups of 25-30 (32%) and 31-35 (30%). The somatic symptoms had a negative association with Christians (p<0.05), whereas they were positively associated with women living in nuclear families (p<0.05) and marriages of less than 3 years (p<0.05). Among the four domains of WHOQOL-BREF, the highest QoL was seen in the social domain (61.06 ± 18.58), the lowest was seen in the psychological domain (54.78 ± 13.05).

Conclusion: Women who have a distant relationship with their husbands manifest somatic symptoms which decrease their quality of life. This emphasizes the need to use different approaches during hospital visits and community-oriented programs to identify and improve mental health among married women and wellbeing in the family.

## Introduction

Women are an important element in our society. Her role keeps on changing. She is a daughter, wife, mother, and so on. Physical separation usually occurs as a result of the husband's occupational commitments. In south India, there is an increasing number of married men who are employed abroad and who do not take their families with them [1]. Most of these so-called "Gulf wives" experienced extreme loneliness. It would be reasonable to expect that the absence of a husband has a deep impact on women’s lives [2].

In the Indian context, two areas seem to be particularly affected: first, in the husbands’ absence, women may have a greater role in family decision-making; second, women may need to fill in for absent husbands in many ways, including care of the home. Women generally report more bodily distress and more numerous, more intense, and more frequent somatic symptoms than men [3].

Studies reporting a sex difference among subjects with somatization have constantly found a clear female dominance [4]. Women have a significantly higher somatization score and a higher number of somatic symptoms than men. Women had an odds ratio (OR) of 1.49 to develop severe somatization symptoms [5]. Astoundingly, in one study of 1000 patients presenting over three years, 567 new complaints of 14 common symptoms were identified [6].

Women are emotionally more expressive and responsive, thus more cases of somatic symptoms are seen in women [7]. Somatic complaints are the cause of up to half of all primary care visits and result in increased medical care expenditures, utilization of health care resources, and disability, and inevitably reduce the quality of life (QoL). Women who report their psychological problems refuse to accept their situation and prefer to keep doctor shopping to such an extent that they discontinue the treatment and become chronic clients. Recognition of abnormal illness behavior in somatoform disorders is important to avoid unnecessary tests and inappropriate treatment and to prevent encouragement and reinforcement of abnormal behavior. Mangalore shares a border with Kerala, the state with the highest remittance in India, which has an influence on the people of Mangalore who fly abroad. Very few research studies have been undertaken on married women with distant relationships [8-10], and only one study has been conducted in India [1]. Hence, the researcher aimed to find the prevalence of somatic symptoms and quality of life among married women having a distant relationship with their spouses.

## Materials and methods

Study population and setting

A descriptive cross-sectional study was conducted between January 2018 and March 2018 in an urban community in Mangalore, Karnataka. The inclusion criteria were married women in the age group of 20-50 years and having a distant relationship with their spouses for more than six months for the purpose of employment. There was no study specifically done to assess somatic symptoms in women with distant relationships. Hence, the researcher conducted a pilot study among a small group of 15 women fulfilling the inclusion criteria, which revealed a prevalence rate of 85% for somatic symptoms. This was used to calculate sample size using Open Epi, keeping an absolute precision of 6%. The calculated sample size was 99; therefore, a total of 100 samples were determined.

Data collection tools

Sociodemographic characteristics were obtained from a self-reported questionnaire including 10 items, namely age, religion, education, occupation, monthly income, type of family, social support, duration of married life, decision-maker in the family, and duration of staying away from the husband.

Measurement of Somatic Symptoms

The scale for the assessment of somatic symptoms (SASS) consists of 20 items. The scale has four subscales, namely, pain-related symptoms, sensory-somatic symptoms, nonspecific somatic symptoms, and biological function-related symptoms. The severity of somatic symptoms ranged from 0 to 3; 0 = absent, 1 = mild, 2 = moderate (interferes with sleep and appetite), and 3 = severe (interferes with sleep, activity, occupation, and social functions) [11]. The SASS has good reliability in both internal consistency (0.98) and the interrater method (0.84). Further, the tool was translated into Kannada, and reliability was checked using Cronbach’s alpha method and found to be 0.83, which shows the tool was reliable [12]. As it is a descriptive tool, no specificity or sensitivity was established in the original tool (Appendices Table 4).

Measurement of QoL

The World Health Organization Quality of Life Scale (WHOQOL)-BREF is a short version of the WHOQOL-100 questionnaire, comprising 26 items. The four domains of WHOQOL BREF are physical (domain 1), psychological (domain 2), social (domain 3), and environmental (domain 4). Two questions (q1 and q2) assess the overall quality of life and general health, respectively. The four domain scores denote an individual’s perception of quality of life in each particular domain [13]. In this study, the English and Kannada versions of WHOQOL-BREF were used, and both versions demonstrated good reliability (<0.70) in all four domains of WHOQOL-BREF (Appendices Table 5).

Data collection procedure

Ethical clearance was obtained from the Institutional Ethical Committee with ref no. AJEC/REV/33/2017. Formal written permission was obtained from the District Health Officer to conduct the research study in an urban community. The information about the research was explained to the participants by the researcher using a participant information sheet and informed consent was taken before data collection and assured that confidentiality would be maintained. Using the snowball sampling technique, 350 families from urban areas of Mangalore (Kunjathbail, Bikarnakatte, Gurupura) were screened and 100 married women having a distant relationship with their spouses were selected for the study. The questionnaire, consisting of sociodemographic variables, the SASS questionnaire, and WHOQOL-BREF, was administered to all participants selected for the study.

Statistical analysis

Descriptive statistics were used to describe the demographic characteristics and outcome variables. The SASS questionnaire was analyzed for the presence of somatoform symptoms and significant somatoform symptoms (rated >3 or severe). The WHOQOL BREF was analyzed for QoL in four domains and overall health. Using the WHOQOL BREF manual, the raw score was transformed onto a scale ranging from 0 to 100. The mean score of each domain and the total were calculated. Higher scores denote higher QoL and vice versa. Then, Spearman's rho was used to calculate the correlation coefficient between the SASS and WHOQOL BREF domains. Multilinear regression was used to identify the predictors of the quality of life and somatic symptoms for married women having a distant relationship with their spouse. All the statistical analyses above were done with SPSS (version 17, IBM Corp., Armonk, NY) and a p-value of <0.05.

## Results

Description of sample characteristics

In the present study, a total of 100 women participated in the study. 32% were in the age group of 26-30 years, and the mean age group was 32.15 years. The majority of the participants were Christians (63%), graduates (38%), working as housewives (43%), and had a family income of >15000 (62%). The majority (80%) of women received support from family members. 48% of women were married for more than five years. Whereas, 28% of women have been living in a distant relationship with their spouse for more than five years.

Prevalence of somatic symptoms and quality of life among married women with a distant relationship

The data revealed that all the participants (100%) experienced at least one somatic symptom with a mild severity level. The number of symptoms experienced ranged between 2 and 20, with an average of 11 symptoms per person. Data revealed that 82% of women experienced at least one symptom at a moderate level, whereas the prevalence of somatic symptoms was 34% (at least one symptom at a severe level). Among these 34 women, the majority were in the age groups of 25-30 (32%) and 31-35 (30%). The data also revealed that 36% experienced five or more somatic symptoms at moderate to severe levels. When analyzed for individual symptoms, it revealed that headache and backache were the most common and diarrhea was the least common somatic symptom among women with distant relationships (Table 1).

**Table 1:** Frequency distribution of married women according to the severity of somatic symptoms

Sl no.	Symptoms	Frequency of severity			
Pain-related symptoms		0	1	2	3
1	Headache	12	45	32	11
2	Backache	18	36	27	9
3	Pain in extremities	41	38	17	4
4	Abdominal pain	54	32	12	2
5	Whole-body ache	48	30	20	2
Sensory somatic symptoms					
6	Tingling, numbness	54	34	11	1
7	Heat & cold sensations	49	35	14	2
8	Palpitations	42	44	14	2
9	Sensation of “gas,” bloating	35	37	20	8
10	Burning sensation	47	31	20	2
Non-specific somatic symptoms					
11	Weakness of body	36	35	23	6
12	Weakness of mind	49	33	13	5
13	Giddiness, dizziness, fainting	49	33	14	4
14	Trembling, tremors	56	31	11	2
15	Tiredness, lethargy	39	36	19	6
Biological function-related symptoms					
16	Lack of sleep	38	38	20	4
17	Lack of appetite	49	36	13	2
18	Lack of libido	57	25	16	2
19	Constipation	25	50	23	2
20	Diarrhea	88	11	1	0

**Figure 1:**
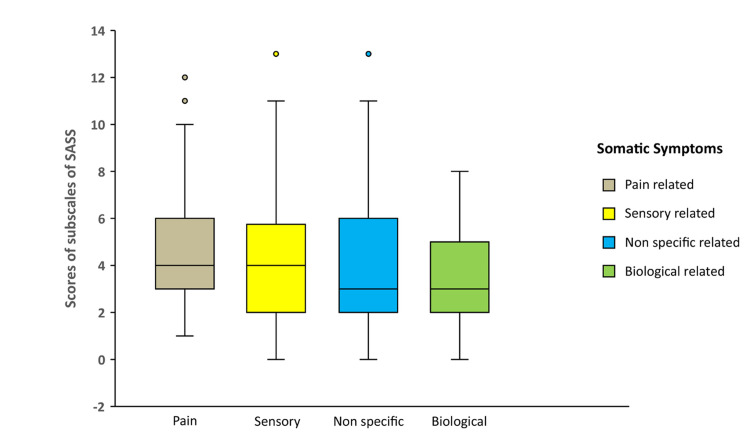
Box plot representing the subscales of somatic symptoms among women with a distant relationship with their spouse

Descriptive analysis of SASS subscales revealed that (Figure 1) the highest score was seen in pain-related symptoms with a median of 4 (IQR 6-3) and the lowest was seen in biologically related symptoms with a median of 3 (IQR 5-2).

**Figure 2:**
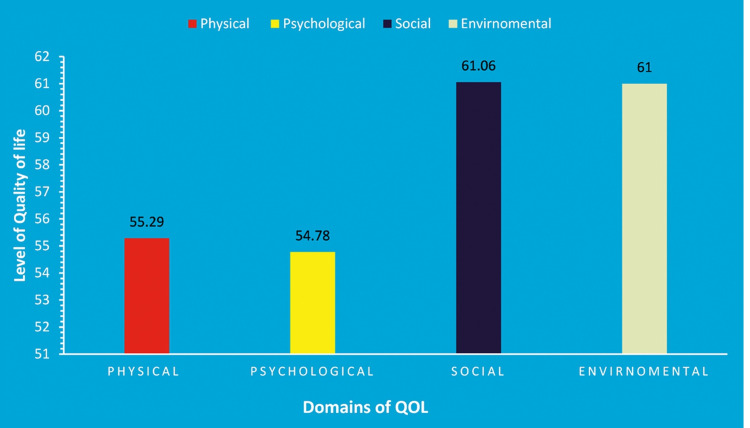
Quality of life of women with a distant relationship in various domains

The quality of life of married women with a distant relationship with their husband is highest in the social domain (61.06 ± 18.58), followed by the environmental domain (61 + 13.74) and the physical domain (55.29 + 10.54), whereas the lowest was seen in the psychological domain (54.78 + 13.05; Figure 2).

According to the data in Table 2, there is a negative correlation between somatic symptoms and all four domains of quality of life, but only the physical domain (r = 0.26, p<0.001) has a significant negative correlation with SASS scores. This shows that women who have a distant relationship with their husbands manifest somatic symptoms that decrease their quality of life. Further analysis found that all four domains of QoL were significantly and positively interrelated with low to moderate correlation (r= 0.35-0.65, p<0.001).

**Table 2:** The correlation coefficient between the somatic symptoms and various domains of quality of life (BREF) among married women having a distant relationship with husbands *p<0.01, **p<0.001

	Mean + SD	SASS	Physical QoL	Psychological QoL	Social QoL	Environmental QoL
SASS	16.17 + 8.62	1	−0.26**	−0.12	−0.11	−0.16
Physical QoL	55.29 + 10.54		1	0.50**	0.50**	0.47**
Psychological QoL	54.78 + 13.05			1	0.53**	0.59**
Social QoL	61.06 + 18.58				1	0.65**
Environmental QoL	61.00 + 13.74					1

Multiple regressions were run to predict the social QoL from income, decision-maker, and duration of the distant relationship. These variables statistically significantly predicted quality of life, F (4.95) = 4.83, p<0.01, R2 = 0.10 (Table 3). Except for the decision-maker, the other two variables added statistically significantly to the prediction, p<0.05. Whereas, multiple regression was run to predict environmental QoL from age, occupation, social support, and duration of the distant relationship. These variables statistically significantly predicted environmental QoL, F (4.95) = 4.92, p<0.01, R2 = 0.13. Except for age, all three remaining variables added statistically significantly to the prediction, p<0.05. Further, multiple regression was run to predict somatic symptoms from age, religion, type of family, and duration of married life and revealed that these variables statistically significantly predicted somatic symptoms, F (4.95) = 4.08, p<0.01, R2 = 0.11 (Table 3). Three variables, except for age, added statistically significantly to the prediction, p<0.05.

**Table 3:** Multilinear regression predicting somatic symptoms, social and environmental quality of life among married women having a distant relationship with their spouses *p<0.05, **p<0.01, ^†^df = (4.95)

Outcome variable	Variables (reference category)	Unstandardized coefficient (β + SE)	F-value^†^	Adjusted R^2^
Social QoL	Income (<15,000)	0.90 + 0.42*	4.83**	0.10
	Decision maker (other than husband)	−0.72 + 0.42		
	Duration of distant relationship (<2 years)	0.95 + 0.42*		
Environmental QoL	Age (>30 years)	−1.71 + 0.92	4.92**	0.13
	Occupation (housewife)	2.21 + 0.91*		
	Social support (yes)	−2. 06 + 0.85*		
	Duration of distant relationship (<2 years)	2.44 + 0.83*		
Somatic symptoms	Age (>30 years)	−3.29 + 1.89	4.08**	0.11
	Religion (other than Christians)	−3.71 + 1.71*		
	Type of family (joint)	3.94 + 1.72*		
	Duration of married life (>3years)	4.86 + 2.00*		

## Discussion

There is a paucity of literature discussing the somatic symptoms and quality of life of married women residing temporarily away from their husbands. The present study attempted to look at the assessment of somatic symptoms and quality of life among married women having a distant relationship with their spouses. Major findings of the study are: (i) 34% of the participants had somatic symptoms at a severe level; (ii) the majority of the women having somatic symptoms were below 30 years of age; (iii) all domains of QoL were negatively correlated to somatic symptoms; (iv) somatic symptoms were more common among women living in nuclear families and married for less than two years; whereas, Christians had fewer chances of having somatic symptoms compared to other communities; and (v) the most common somatic symptoms were headache, back pain, and a sensation of gas bloating.

In the present study, the subscale of pain-related symptoms was manifested most frequently among married women in comparison to the other subscales of somatic symptoms. The findings of the current study were consistent with findings conducted in Karnataka, India, where the majority (88.6%) of the samples had a headache as a common symptom, followed by dizziness (51.9%) and back pain (46.8%) [14]. The study implies that women living alone have to deal with numerous hardships and loneliness. Though family support is evident, the absence of their husband is felt. When such feelings are developed, somatization symptoms prevail. It is the woman's expression of the need for love and attention from the husband as well as family members.

The proportion of married women having somatic symptoms found in the current study was higher than in Hyderabad (14%) [1]. But, similar to the prevalence of somatic symptoms (40.8%) found in Kerala [15]. Previous studies from India [7] have also stated that married women are more prone to somatic symptoms and medically unexplained physical symptoms (MUPS). All this emphasizes the need to check the psychological aspect when treating married women who are having somatic complaints. The need to look into family issues like a distant relationship can help reduce the problem of somatic symptoms.

In the present study, the QoL in various domains was a reflection of study findings from India [16], which reported that QoL in the social domain was the highest (60.24 + 15.03) and the lowest was seen in the physical domain (55.87 + 13.64), whereas a study in Turkey [17] reported a higher mean score in physical (61.98 + 10.08), psychological (66.32 + 10.47), social (67.12 + 11.92) and environmental (69.60 + 10.23) among pregnant women with OCD. Another study in China [10] revealed that women left behind had lower QoL than non-left-behind women. All the above studies state that women with distant relationships have lower QoL than other women.

In the current study, the correlation between domains of QoL was low to a moderate level and was positively significant. Whereas a study conducted in China [18] revealed a correlation with a very low to low level of correlation (r = 0.01-0.48) and the relationship between environmental and physical/social was not significant, all other relationships between the domains were significant (p<0.05). The result reveals that in the current study, all the domains of QoL are unidirectional and positively related to each other.

In the present study, social QoL had a significant positive association with income and a negative association with the duration of a distant relationship. Whereas, environmental QoL had a positive association with occupation and a negative association with the duration of distant relationships (Table 2), which was consistent with the finding of a study from China [18]. On the contrary, social support is closely related to financial status or income. The higher the income, the higher the social QoL. However, there is a negative association between social and environmental QoL and the duration of distant relationship states. As the duration increases, the QoL decreases. It may be related to increasing responsibilities like children and the care of elderly family members. Increasing age may even cause a decreased sense of marriage security [18]. Occupation has a positive association with environmental QoL. This may be because occupation gives a sense of financial freedom and also improves the sense of well-being. Having a job can make a lot of difference for women, especially in a distant relationship.

In the current study (Table 3), somatic symptoms have a significant negative association with religion and duration of the marriage, whereas they are positively associated with the type of family. The results were in parallel with a study conducted in India [7]. Another study from India [19] has found that living with family acts as a protective factor against somatic symptoms. All these results state that living alone or as a nuclear family has a high chance of having somatic symptoms. This may be because loneliness leads to all kinds of psychiatric problems, including somatic symptoms, and nuclear families have decreased social support compared to joint families. Whereas from a religious point of view, Christianity has a significant negative association. This may be their way of living or they may have adjusted and seen a similar kind of life with their parents.

Studies have been done on somatic symptoms and have revealed that women are more prone to somatic symptoms [19]. But in some places, the severity is greater, like in Kerala [15] and Mangalore. The researcher has tried to identify one specific population/sub-group which may be more prone to such somatic symptoms than other married women, i.e., married women with a distant relationship. Future research can be done on a larger population so that generalization can be done.

The study has limitations, such as the number of respondents included, which is less even after major efforts. The sampling was done using snowball sampling. The study was conducted only in the selected urban community, which imposed limits on the generalization of findings to another setting or cities with fewer women having distant relationships. Only subjective responses were taken into consideration. No direct observation was done to assess the symptoms of women having a distant relationship with their husbands.

## Conclusions

Married women having a distant relationship have a high prevalence of somatic symptoms, which further leads to a lower quality of life. It emphasizes the need for a different approach to deal with such a population. Efforts have to be made to improve mental health, social support, and autonomy among married women, especially during the initial years of marriage, which will assist in adaptation to the new environment and family. Physical problems like headaches and back pain which are persistent for months should be handled with the help of professionals to rule out the presence of somatic symptoms. Health care professionals like physicians, obstetricians, gynecologists, and community health workers need to identify the risk population and prompt referrals for mental health consultation. Occupation and family should go hand in hand and should not cause negative effects on one another, as both are essential parts of everyone's day-to-day life.

## Appendices

**Table 4:** Scale for assessment of somatic symptoms The severity of SS is rated from 1 to 3. (1: mild; 2: moderate - interferes with sleep, appetite; 3: severe - interferes with sleep, activity, occupation, and social functions). The SS is to be considered as the present should have been noted or occurred during the previous two weeks.

Sl no	Symptoms	Severity			
Pain-related symptoms		0	1	2	3
1	Headache				
2	Backache				
3	Pain in extremities				
4	Abdominal pain				
5	Whole-body ache				
Sensory somatic symptoms					
6	Tingling, numbness				
7	Heat and cold sensations				
8	Palpitations				
9	Sensation of “gas,” bloating				
10	Burning sensation				
Non-specific somatic symptoms					
11	Weakness of body				
12	Weakness of mind				
13	Giddiness, dizziness, fainting				
14	Trembling, tremors				
15	Tiredness, lethargy				
Biological function-related symptoms					
16	Lack of sleep				
17	Lack of appetite				
18	Lack of libido				
19	Constipation				
20	Diarrhea				

**Table 5:** WHOQOL-BREF The following questions ask how you feel about your quality of life, health, or other areas of your life. I will read out each question to you, along with the response options. Please choose the answer that appears most appropriate. If you are unsure about which response to give to a question, the first response you think of is often the best one. Please keep in mind your standards, hopes, pleasures and concerns. We ask that you think about your life in the last four weeks.

		Very poor	Poor	Neither poor nor good	Good	Very good
1	How would you rate your quality of life?	1	2	3	4	5
		Very dissatisfied	Dissatisfied	Neither satisfied nor dissatisfied	Satisfied	Very satisfied
2	How satisfied are you with your health?	1	2	3	4	5
		Not at all	A little	A moderate amount	Very much	An extreme amount
3	To what extent do you feel that physical pain prevents you from doing what you need to do	5	4	3	2	1
4	How much do you need any medical treatment to function in your daily life?	5	4	3	2	1
5	How much do you enjoy life?	1	2	3	4	5
6	To what extent do you feel your life is meaningful?	1	2	3	4	5
		Not at all	A little	A moderate amount	Very much	extremely
7	How well are you able to concentrate?	1	2	3	4	5
8	How safe do you feel in your daily life?	1	2	3	4	5
9	How healthy is your physical environment?	1	2	3	4	5
		Not at all	A little	moderately	mostly	completely
10	Do you have enough energy for everyday life?	1	2	3	4	5
11	Are you able to accept your bodily appearance?	1	2	3	4	5
12	Have your enough money to meet your needs?	1	2	3	4	5
13	How available to you is the information that you need in your day to day life?	1	2	3	4	5
14	To what extent do you have opportunities for leisure activities?	1	2	3	4	5
		Very poor	Poor	Neither poor nor good	Good	Very good
15	How well are you able to get around?	1	2	3	4	5
		Very dissatisfied	Dissatisfied	Neither satisfied nor dissatisfied	satisfied	Very satisfied
16	How satisfied are you with your sleep?	1	2	3	4	5
17	How satisfied are you with your ability to perform your daily living activities?	1	2	3	4	5
18	How satisfied are you with your capacity to work?	1	2	3	4	5
19	How satisfied are you with yourself?	1	2	3	4	5
20	How satisfied are you with your personal relationships?	1	2	3	4	5
21	How satisfied are you with your sex-life?	1	2	3	4	5
22	How satisfied are you with the support you get from your friends?	1	2	3	4	5
23	How satisfied are you with the conditions of your living place?	1	2	3	4	5
24	How satisfied are you with your access to health services?	1	2	3	4	5
25	How satisfied are you with the transport facility?	1	2	3	4	5
		Never	Seldom	Quite often	Very often	Always
26	How often do you have negative feelings such as blue-mood, despair, anxiety, depression?	5	4	3	2	1
